# Investigating the Molecular Mechanisms of Renal Hepcidin Induction and Protection upon Hemoglobin-Induced Acute Kidney Injury

**DOI:** 10.3390/ijms23031352

**Published:** 2022-01-25

**Authors:** Laura E. Diepeveen, Gaby Stegemann, Erwin T. Wiegerinck, Rian Roelofs, Myrthe Naber, Olivier Lóreal, Bart Smeets, Frank Thévenod, Dorine W. Swinkels, Rachel P. L. van Swelm

**Affiliations:** 1 Translational Metabolic Laboratory (TML-830), Department of Laboratory Medicine, Radboud Institute for Molecular Life Sciences, Radboud University Medical Center, 6525 GA Nijmegen, The Netherlands; Laura.Diepeveen@radboudumc.nl (L.E.D.); gabystegemann@hotmail.com (G.S.); erwin.wiegerinck@radboudumc.nl (E.T.W.); rian.roelofs@radboudumc.nl (R.R.); mhnaber@gmail.com (M.N.); dorine.swinkels@radboudumc.nl (D.W.S.); 2 INSERM 1241, Nutrition, Metabolisms and Cancer (NuMeCan) Institute, University of Rennes, 35033 Rennes, France; olivier.loreal@univ-rennes1.fr; 3 Department of Pathology, Radboud Institute for Molecular Life Sciences, Radboud University Medical Center, 6525 GA Nijmegen, The Netherlands; Bart.Smeets@radboudumc.nl; 4 Institute of Physiology, Pathophysiology and Toxicology, Center for Biomedical Training and Research (ZBAF), University of Witten/Herdecke, 58455 Witten, Germany; frank.thevenod@uni-wh.de

**Keywords:** kidney, iron, hepcidin, acute kidney injury, hemoglobin

## Abstract

Hemolysis is known to cause acute kidney injury (AKI). The iron regulatory hormone hepcidin, produced by renal distal tubules, is suggested to exert a renoprotective role during this pathology. We aimed to elucidate the molecular mechanisms of renal hepcidin synthesis and its protection against hemoglobin-induced AKI. In contrast to known hepatic hepcidin induction, incubation of mouse cortical collecting duct (mCCD_cl1_) cells with IL-6 or LPS did not induce *Hamp1* mRNA expression, whereas iron (FeS) and hemin significantly induced hepcidin synthesis (*p* < 0.05). Moreover, iron/heme-mediated hepcidin induction in mCCD_cl1_ cells was caused by the nuclear factor erythroid 2-related factor 2 (Nrf2) pathway, as indicated by increased nuclear Nrf2 translocation and induced expression of Nrf2 downstream targets GCLM (*p* < 0.001), NQO1 (*p* < 0.001), and TXNRD1 (*p* < 0.005), which could be prevented by the known Nrf2 inhibitor trigonelline. Newly created inducible kidney-specific hepcidin KO mice demonstrated a significant reduction in renal *Hamp1* mRNA expression. Phenylhydrazine (PHZ)-induced hemolysis caused renal iron loading and oxidative stress in both wildtype (Wt) and KO mice. PHZ treatment in Wt induced inflammatory markers (*IL-6*, *TNFα*) but not *Hamp1*. However, since PHZ treatment also significantly reduced systemic hepcidin levels in both Wt and KO mice (both *p* < 0.001), a dissection between the roles of systemic and renal hepcidin could not be made. Combined, the results of our study indicate that there are kidney-specific mechanisms in hepcidin regulation, as indicated by the dominant role of iron and not inflammation as an inducer of renal hepcidin, but also emphasize the complex interplay of various iron regulatory mechanisms during AKI on a local and systemic level.

## 1. Introduction

Major surgery with cardiopulmonary bypass (CPB) is the second most common cause of life-threatening acute kidney injury (AKI), after sepsis [1]. AKI as a postoperative complication affects up to one-third of patients and is associated with increased mortality and morbidity. It has been recognized that an important contribution to postoperative AKI is made by hemolysis as a result of the mechanical forces of both the blood pump and suction system during CPB [2,3]. Disruption of circulating red blood cells (RBCs) leads to excessive release of hemoglobin into the bloodstream [4,5]. Upon saturation of the systemic hemoglobin scavenger haptoglobin, hemoglobin and its toxic reactive breakdown products heme and free iron are filtered by the glomerulus [5,6]. Hemoglobin can be reabsorbed in the proximal tubules through the megalin/cubulin complex via endocytosis [7] and in distal tubules by the neutrophil gelatinase-associated lipocalin (NGAL) receptor [8]. Intracellularly, hemoglobin dissociates into heme and globin under oxidant conditions. Heme is subsequently catabolized by heme oxygenase-1 (HO-1) into billiverdin, CO, and reactive iron (Fe^2+^). The reactive iron is converted to Fe^3+^ by H-Ferritin and thereafter stored by L-Ferritin or exported intro the blood by the only known mammalian cellular iron exporter, ferroportin [9,10]. However, during severe hemolysis, hemoglobin, heme, and free iron may accumulate in the kidney, promoting renal cell damage [11]. The exact mechanisms underlying hemoglobin-induced AKI have not been completely elucidated but appear to be multi-factorial and intertwined. To date, there are no adequate measures to prevent or treat hemoglobin-induced AKI. 

Clinical observations describe an association between high urinary levels of the iron-regulatory hormone hepcidin and a reduced risk of AKI in patients undergoing cardiac surgery, suggesting a possible protective effect of hepcidin [12,13,14] Since these patients presented with similar hepcidin plasma concentrations, the increased urinary levels of hepcidin could be due to locally produced renal hepcidin. Indeed, hepcidin is shown to be synthesized in the distal tubules of the kidney [8,15,16]. In vivo studies strengthened the idea of hepcidin exerting a renoprotective effect, since renal damage was shown to be reduced upon intraperitoneal administration of hepcidin during both ischemia–reperfusion injury and hemoglobin-induced injury [15,17]. Whereas exogenously administered hepcidin was shown to be reabsorbed by proximal tubule cells [15], hemoglobin-induced injury also increased distal nephron hepcidin synthesis [8,15]. These findings suggest that both locally produced and systemic hepcidin might contribute to the observed protective effect. A role for renal hepcidin was confirmed in in vitro studies where silencing of hepcidin in mouse cortical collecting duct cells (mCCD_cl1_) aggravated oxidative, inflammatory, and endoplasmic reticulum (ER) stress after hemoglobin incubation [8]. Interestingly, hemoglobin and hemin triggered induction of *HO-1* and *interleukin-6 (IL-6),* in vivo and in cultured mCCD_cl1_ cells [8,15], suggesting renal hepcidin upregulation by upstream mechanisms involving both iron/hemin-dependent oxidative and inflammatory signaling. However, hepcidin mRNA (*Hamp1*) silencing in mCCD_cl1_ cells abolished basal IL-6 expression and exacerbated HO-1 and IL-6 expression induced by hemoglobin and hemin, hinting at complex downstream roles of hepcidin in hemoglobin-induced renal injury [8]. Nevertheless, the exact molecular cascade of renal hepcidin synthesis and subsequent renoprotective function have not yet been unraveled.

Here, we studied the mechanism of hepcidin induction in mCCD_cl1_ cells in the context of hemin exposure, the iron-containing component of heme [8]. Moreover, we set out to determine the contribution of renal hepcidin to protection against hemoglobin-induced kidney injury in vivo using newly developed kidney-specific inducible hepcidin knockout (KO) mice. Gaining insight into these pathways is a first step in exploring the therapeutic options of hepcidin for AKI.

## 2. Results

### 2.1. Iron Rather Than Inflammation Induces Hepcidin Synthesis

**Figure 1:**
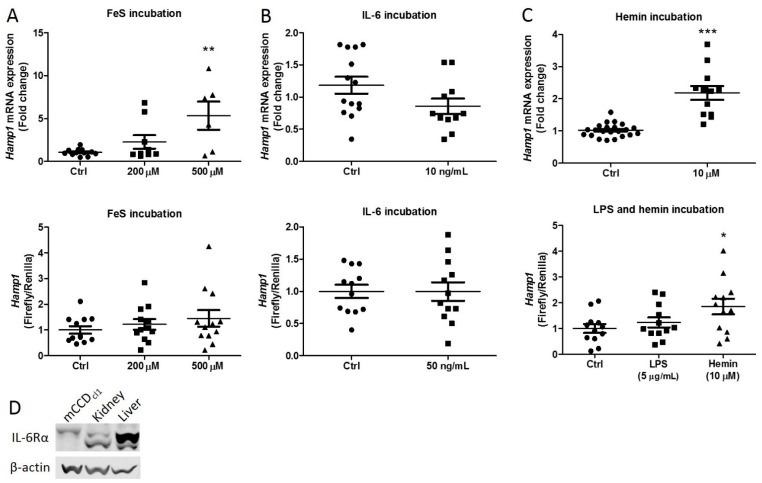
The effect of inflammation and iron on hepcidin expression in mCCD_cl1_ cells. Hepcidin mRNA expression and luciferase activity after (**A**) 4 h of IL-6 incubation, (**B**) 16 h of FeS incubation, and (**C**) 4 h of hemin and LPS incubation. PBS was used as a control. (**D**) Western blot image of the IL-6 receptor (IL-6Rα) in mCCD_cl1_ lysate, mouse kidney, and mouse liver homogenate. Graphs A to C show the mean ± SEM of at least three independent experiments in duplicate. Circles, squares and triangles show individual data points in the indicated treatment group. * *p* < 0.05, ** *p* < 0.01, *** *p* < 0.001 compared to the control using a one-way ANOVA with Bonferroni’s multiple comparisons test or a *t*-test.

Incubation of mCCD_cl1_ cells with hemin previously resulted in increased intracellular iron and inflammation [8], both of which can induce systemic hepcidin synthesis in the liver [18,19]. To determine what drives renal hepcidin synthesis during heme-mediated AKI, we incubated mCCD_cl1_ cells with the inflammatory cytokine IL-6 and iron sulphide (FeS), which releases ferrous iron upon dissolving. Surprisingly, hepcidin synthesis was not increased upon IL-6 incubation as indicated by both the *Hamp1* mRNA expression levels and luciferase activity in cells transfected with a luciferase construct coupled to a *Hamp* promotor (Figure 1A). FeS, on the other hand, showed a dose–response trend in hepcidin induction using both qPCR and the luciferase assay (Figure 1B). These results suggest that iron rather than inflammation initiates hepcidin synthesis in distal tubule cells. These findings were strengthened by increased hepcidin induction upon incubation with hemin but not with the inflammation inducer LPS (Figure 1C). The presence of the IL-6 receptor (IL-6Rα) was confirmed with Western blot in untreated mCCD_cl1_ lysates (Figure 1D). The IL-6R was present in mCCD_cl1_ cells, although to a lesser extent than compared to control mouse kidney or liver tissue (Figure 1D). 

### 2.2. Downregulation of Hemin-Induced Nrf2 Pathway Activation Decreases Hepcidin Synthesis

The nuclear factor erythroid 2-related factor 2 (Nrf2) pathway, a mediator of cellular stress defense [20], can modulate systemic hepcidin synthesis in the liver [19,21,22]. Interestingly, it has been shown that iron exposure activates the Nrf2 pathway in proximal tubule cells [23] and that the iron in hemin induces hepcidin synthesis. Therefore, we investigated whether hemin-induced renal hepcidin expression is mediated by Nrf2 in mCCD_cl1_ cells and, subsequently, assessed whether Nrf2 pathway modulation affects hepcidin synthesis. To this end, we used the Nrf2 inhibitor trigonelline [23].

**Figure 2:**
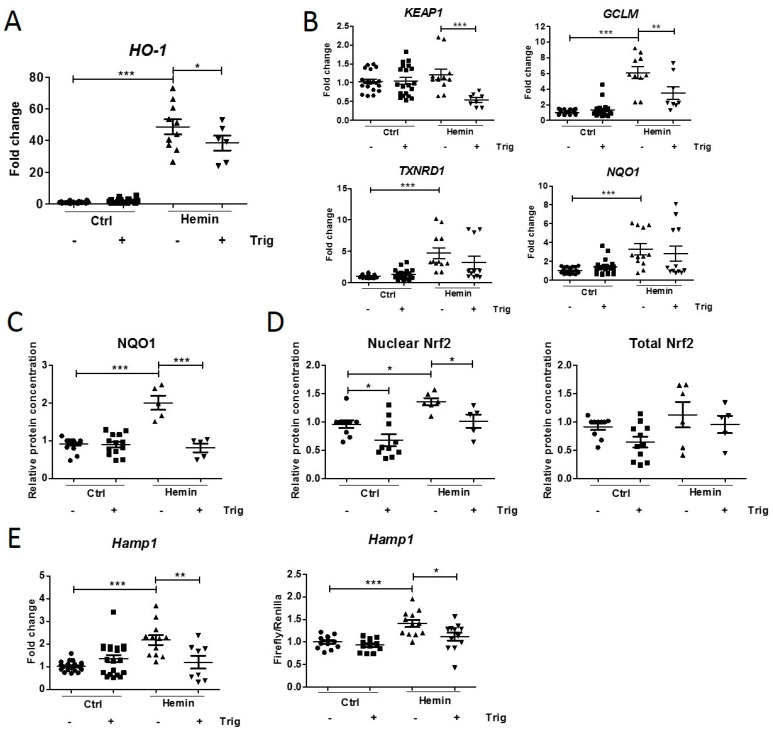
Hemin-induced Nrf2 pathway activation and subsequent downregulation using trigonelline in mCCD_cl1_ cells. (**A**) *HO-1*, (**B**) *KEAP1*, *GCLM*, *TXNRD1*, *NQO1* mRNA expression, and (**C**) nuclear Nrf2, total cellular Nrf2, and (**D**) NQO1 protein concentrations after 4 h of 10 µM hemin incubation with or without 1 µM trigonelline (trig). (**E**) *Hamp1* mRNA expression and hepcidin synthesis in mCCD_cl1_ cells after 10 μM hemin with or without 1 μM trigonelline for 4 h. PBS was used as a control. Protein concentrations were relative to either histone H3 (nuclear fraction) or β-actin (total cell lysate). Representative images and graphs (mean ± SEM) show the mean of three independent experiments. Circles, squares and triangles show individual data points in the indicated treatment group. * *p* < 0.05, ** *p* < 0.01, *** *p* < 0.001 compared to the control using a one-way ANOVA with Bonferroni’s multiple comparisons test or a *t*-test.

Hemin incubation increased *HO-1* mRNA expression compared to the control, indicating heme catabolization by the mCCD_cl1_ cells and oxidative stress occurred [24], which decreased upon co-incubation with trigonelline (Figure 2A). Accordingly, Nrf2 pathway activation upon hemin incubation was observed by significantly increased mRNA expression of the Nrf2 target genes *thioredoxin reductase 1* (*TXNRD1*), *glutamate-cysteine ligase modifier subunit (GCLM*), and *NAD(P)H quinone dehydrogenase 1* (*NQO1*) and protein concentrations of NQO1 (Figure 2B,C). Trigonelline, in turn, significantly reduced *Kelch-like ECH-associated protein 1* (*KEAP1*) and *GCLM* mRNA and NQO1 protein expression (Figure 2B,C). Because Nrf2 is a transcription factor and functions by translocating from the cytoplasma to the nucleus [20], nuclear Nrf2 protein levels were additionally studied. Hemin incubation caused an increase in Nrf2 in mCCD_cl1_ nuclear cell lysates compared to controls, without significantly affecting the total cellular Nrf2 protein levels (Figure 2D). Co-incubation with trigonelline reduced Nrf2 translocation, yet this significant reduction was also observed in control cells. Altogether, these data support activation of the Nrf2 pathway upon hemin-induced injury in mCCD_cl1_ cells, which can be inhibited by co-incubation with trigonelline. Interestingly, hemin was found to increase hepcidin mRNA expression and luciferase activity, which was significantly downregulated by reduced Nrf2 activation using trigonelline in mCCD_cl1_ cells (Figure 2E). These observations indicate that hepcidin synthesis occurs through Nrf2 activation in these cells upon hemin-induced injury.

### 2.3. The Effect of a Renal Hepcidin KO

**Figure 3:**
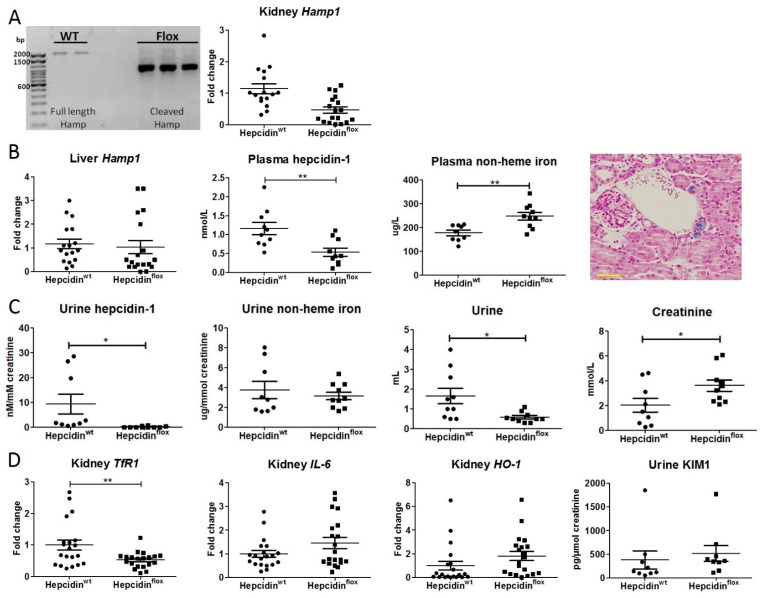
Characterization of renal hepcidin KO in mice. (**A**) PCR gel of renal hepcidin gene amplification and *Hamp1* mRNA expression; (**B**) hepatic *Hamp1* mRNA expression, plasma hepcidin-1 levels, plasma non-heme iron concentration and representative image of Perls iron staining in hepcidin^flox^ kidney; (**C**) urine hepcidin-1 levels, urine non-heme iron concentration, 24 h urine production, and urine creatinine concentration; and (**D**) renal mRNA expression of *TfR1*, *HO-1*, *IL-6*, and urine concentrations of KIM1 of wild-type and hepcidin KO mice (*n* = 10 per group). MRNA expression data is shown in duplicates for each individual mouse. Circles, squares and triangles show individual data points in the indicated treatment group. Graphs show the mean ± SEM. * *p* < 0.05, ** *p* < 0.01 using a *t*-test. Scalebar = 50 µM.

To further assess the regulation of renal hepcidin by iron and inflammation and to study the contribution of renal hepcidin in protection during hemoglobin-induced AKI, inducible and kidney-specific hepcidin knock-out (KO) mice (hepcidin^flox^) were generated. Both wild-type (WT) mice (hepcidin^wt^) and hepcidin^flox^ mice were subjected to doxycycline treatment, which induced hepcidin KO in the hepcidin^flox^ mice. Subsequently, both mice were phenotypically characterized. The renal hepcidin KO was shown to be successful as both the renal hepcidin DNA product and mRNA expression were reduced in hepcidin^flox^ mice (Figure 3A), although the difference in mRNA expression between the hepcidin^wt^ and hepcidin^flox^ mice did not reach statistical significance. Although the liver mRNA expression levels of *Hamp1* in both mice were similar, plasma hepcidin levels were significantly lower in hepcidin^flox^ mice compared to hepcidin^wt^ mice (Figure 3B). In parallel, we observed significantly higher plasma non-heme iron levels in the hepcidin^flox^ mice. Moreover, in the kidney sections of 2 out of 10 hepcidin^flox^ mice, we found isolated tubules with iron deposition (Figure 3B). Whereas the significantly reduced plasma hepcidin-1 levels in hepcidin^flox^ mice were reflected in urine, the increased plasma non-heme iron concentration was not (Figure 3C). Interestingly, hepcidin^flox^ mice produced significantly less urine in 24 h compared to hepcidin^wt^ mice, with a significantly higher creatinine concentration (Figure 3C). We observed a significant reduction of renal transferrin receptor 1 (*TfR1*) mRNA expression in hepcidin^flox^ mice. TfR1 is negatively regulated by intracellular iron and therefore its reduction may indicate an increase in cellular iron [25]. Trends in increased *HO-1* and *IL-6* mRNA levels and urine kidney injury molecule 1 (KIM1) in hepcidin^flox^ mice may also indicate some degree of renal stress (Figure 3D). Together, these data confirm the renal hepcidin ablation in hepcidin^flox^ mice and suggest that renal hepcidin may have a role in physiological renal iron homeostasis. 

**Figure 4:**
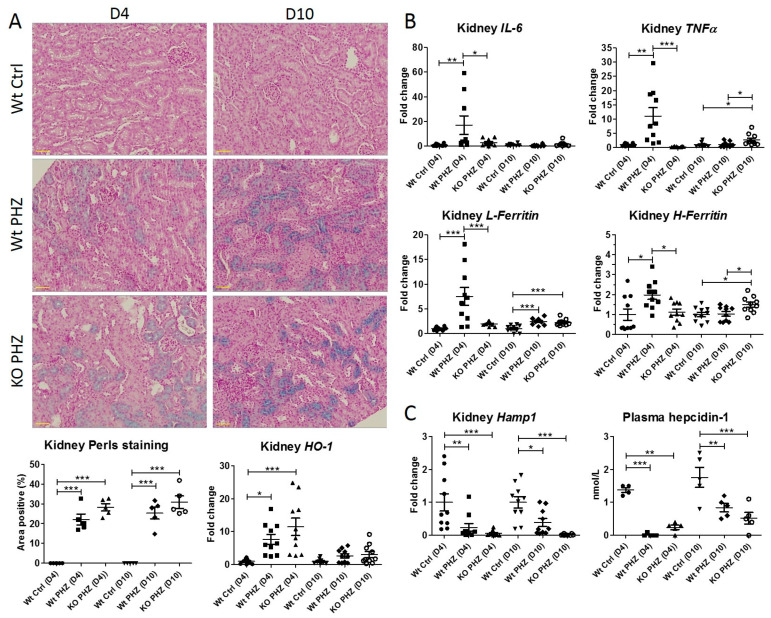
The effect of PHZ in hepcidin KO and Wt mice. (**A**) Perls staining and quantification in kidney sections, in which blue deposition indicates iron, and renal mRNA expression of *HO-1,* (**B**) *IL-6*, *TNFα*, *L-Ferritin*, *H-Ferritin* (**C**) *Hamp1*, and plasma hepcidin-1 concentrations. *n* = 5 mice for each group, sacrificed at day 4 (D4) or day 10 (D10) after PHZ injection to induce hemolysis. Circles, squares and triangles show individual data points in the indicated treatment group * *p* < 0.05, ** *p* < 0.01, *** *p* < 0.001 using a one-way ANOVA with Bonferroni’s multiple comparisons test or a *t*-test. Scalebar = 40 µM.

Doxycyline-treated hepcidin^wt^ and hepcidin^flox^ mice were subjected to phenylhydrazine (PHZ) to induce hemolysis and were sacrificed after 4 (D4) or 10 (D10) days. PHZ-mediated kidney injury was effective since compared to non-PHZ-injected hepcidin^wt^ mice (Wt Ctrl), both PHZ-treated hepcidin^wt^ mice (Wt PHZ) and hepcidin^flox^ mice (KO PHZ) demonstrated clear renal iron accumulation on D4 and D10 using Perls staining (Figure 4A). Moreover, increased renal mRNA expression of *HO-1* for Wt PHZ and KO PHZ mice indicated intracellular hemoglobin catabolization, especially at D4 (Figure 4A). Both iron accumulation and *HO-1* expression appeared to be higher in KO mice compared to Wt mice, albeit not significantly. Next, we measured the mRNA expression of inflammation and intracellular iron markers to assess renal injury and iron regulation, as used in previous studies [8,15]. Strikingly, *IL-6*, *Tumor Necrosis Factor α* (*TNFα*), *L-Ferritin*, and *H-Ferritin* mRNA expression was induced in Wt PHZ mice at D4 but not in KO PHZ mice (Figure 4B). In turn, KO PHZ mice showed significantly increased *TNFα* and *H-Ferritin* mRNA expression at D10 after PHZ and, to a lesser extent, elevated *IL-6* mRNA expression. Kidney *Hamp1* mRNA expression was significantly reduced in the KO PHZ mice compared to Wt Ctrl as expected but surprisingly also in the Wt PHZ mice (Figure 4C) despite *IL-6* upregulation (Figure 4B). Moreover, plasma hepcidin levels in the KO PHZ mice, and even more in the Wt PHZ mice, were also significantly reduced compared to Wt Ctrl on D4 and D10.

## 3. Discussion

Hepcidin has been suggested to exert a renoprotective role during AKI. With this study, we aimed to determine the molecular mechanism of renal hepcidin induction upon hemoglobin-induced injury in vitro and to obtain greater insight into the protective contribution of renal hepcidin in vivo.

We found that hemin-induced hepcidin synthesis in mCCD_cl1_ cells was mediated by the Nrf2 pathway. Hemin increased the mRNA expression of Nrf2 target genes *GCLM*, *TXNRD1*, and *NQO1*; protein levels of NQO1; and translocation of Nrf2. The effect of hemin incubation on *KEAP1* expression was less evident, potentially explained by the fact that KEAP1 is not a target gene of Nrf2 but a cytosolic protein regulating Nrf2 translocation [20]. Therefore, increased cellular stress might not have affected its transcription. Despite the absence of a clear reduction of nuclear Nrf2 translocation upon co-incubation of hemin and the Nrf2 inhibitor trigonelline, this condition did significantly reduce the mRNA expression and protein levels of Nrf2 target genes, demonstrating a reduction of the Nrf2 pathway’s activity. Reduced nuclear Nrf2 by trigonelline in control cells did not result in any changes in the target genes, which is likely explained by the fact that under baseline conditions, Nrf2 activity is minimal. Indeed, Nrf2 KO mice have been shown to not be present with any phenotype when unchallenged [26]. 

Our observations of a renoprotective effect of Nrf2 comply with previous studies that showed aggravation of diabetic nephropathy [27] and autoimmune nephritis [28] in mice lacking Nrf2, protection from ischemia-reperfusion injury in mice with tubular hyperactivation of Nrf2 [29], and increased glomerular filtration rates in patients with chronic kidney disease that were treated with an Nrf2 activator [30]. The Nrf2 pathway has also been previously linked to iron signaling, since Nrf2 was found to regulate HO-1 and Ferritin in mice, proteins involved in hemoglobin catabolism and iron storage, respectively [31,32]. Yet, we are the first to show that Nrf2 pathway activation is involved in iron-induced renal hepcidin synthesis. Our findings corroborate earlier observations of an Nrf2-binding site in the *Hamp* promoter region and Nrf2 controlling hepcidin synthesis in hepatocytes during oxidative stress [21,22], which can be caused by an excess of free iron as a result of hemolysis [33]. Indeed, a systemic increase of iron has been shown to lead to the formation of reactive oxygen species (ROS), activating the Nrf2 pathway in liver sinusoidal endothelial cells (LSECs) and hepatocytes. Although Nrf2 is thought to evoke an antioxidant response in both cell types, it is proposed that LSECs excrete bone morphogenic protein 6 (BMP6) that induces hepcidin synthesis in neighboring hepatocytes by paracrine signaling [19]. It might be plausible that BMP6 is produced in the kidney by the Nrf2 pathway during hemin exposure, which in turn mediates the upregulation of hepcidin. Although little is known about BMP6 in the kidney, it is expressed in the renal cortex, predominantly in the tubulo-interstitium containing the proximal and distal epithelial cells, and BMP6 deficiency caused aggravated tubular cell damage in mice [34]. Moreover, the observed paracrine signaling of BMP6 in the liver might offer insights into the dynamics of hemoglobin injury and hepcidin production as observed previously in mice [15,35]. Further research is needed to explore the potential role of BMP6 in Nrf2-induced hepcidin synthesis in distal tubule cells and tubular crosstalk. 

Interestingly, although inflammation is a known potent inducer of hepcidin in hepatocytes via IL-6 signaling [36], *Hamp1* expression was not affected by IL-6 or LPS in distal tubular epithelial cells during 4-h incubations. This was confirmed in our in vivo model, where we observed a PHZ-induced increase in *IL-6* in Wt mice at D4 and a simultaneous reduction in kidney *Hamp1*. Although our molecular insights into kidney iron handling are still limited, this study importantly reveals that the regulation of kidney hepcidin differs from the known canonical signaling described in the liver. Future in-depth molecular studies are warranted to corroborate these findings in more detail. 

We developed inducible kidney-specific hepcidin KO mice to investigate the role of renal hepcidin in protection during hemolysis-induced AKI. The renal hepcidin KO was shown to be successful at reducing kidney hepcidin mRNA expression levels, which were accompanied by alterations in intracellular iron levels and some degree of renal stress. This suggests a physiological relevance for renal hepcidin, which complies with previous in vitro findings, where hepcidin silencing in unchallenged mCCD_cl1_ cells increased oxidative stress [8]. Nevertheless, urine and plasma hepcidin levels were also reduced in KO mice to approximately half that of controls. This could either suggest that renal hepcidin synthesis contributes to systemic levels, or that systemic hepcidin production was affected by the genetic construction. Indeed, it has been demonstrated that approximately 10% of the hepatocytes also express Pax8 [37], and with hepatocytes being the primary site for systemic hepcidin production, it is plausible that the Pax8-driven deletion has affected hepatic hepcidin synthesis. However, it remains unclear whether diminished hepatic synthesis could account for the full extent of the lowered plasma hepcidin levels. In line with reduced circulating plasma hepcidin levels, plasma non-heme iron levels were increased in hepcidin^flox^ mice. The incidental iron deposition observed in some hepcidin^flox^ kidneys, combined with the lack of an increased urine non-heme iron concentration, may suggest that the circulating increased non-heme iron was reabsorbed in the kidney. Nevertheless, potential enhanced iron uptake by other organs was not determined and cannot be disregarded. 

**Figure 5:**
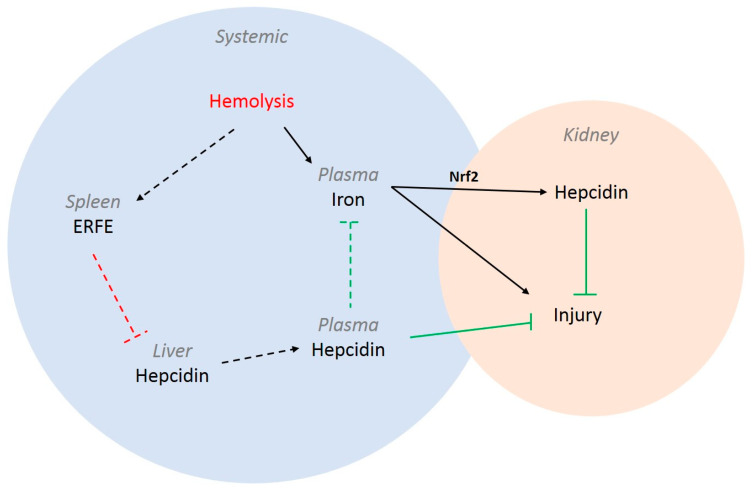
Schematic overview of intertwined systemic and kidney iron handling during hemolysis-induced AKI. Systemic hemolysis results in increased circulating iron levels that are deposited in the kidney, causing acute injury. In parallel, iron can induce kidney hepcidin synthesis through the Nrf2 pathway, which can protect against iron-mediated injury. On a systemic level, hemolysis can activate splenic ERFE production that, in turn, inhibits hepatic hepcidin synthesis, thereby reducing circulating plasma hepcidin levels. Plasma hepcidin can also contribute to kidney protection against injury. Solid lines represent data from this or previous studies on hemolytic kidney injury; dotted arrows represent our hypothesis in the context of hemolysis-mediated AKI. Black lines indicate induction, red lines indicate inhibition, and green lines indicate protection.

Moreover, although the inducible kidney-specific mouse model was designed to specifically dissect the role of renal hepcidin from systemic hepcidin, unfortunately, the PHZ treatment itself influenced systemic hepcidin levels greatly. Hemolysis, caused by the PHZ injection, likely triggered an erythropoietic response by erythroferrone (ERFE), which can reduce systemic hepcidin levels to ensure iron availability for the production of new red blood cells [38]. Glomerular filtered systemic hepcidin can be reabsorbed in the proximal tubules, where it is suggested to protect against iron-mediated injury [15]. Indeed, crosstalk between hepatic and renal iron regulatory systems has been observed, for instance, for hypoxia-inducible factor 2 α (HIF2a), where hepatic HIF2a takes over the function of renal HIF2a when ablated [39]. It is not unlikely that the renal inflammation triggered by hemolysis via IL-6 could induce hepatic *Hamp*1 synthesis to aid in renal protection. However, since it has been observed that ERFE signaling overrules inflammation-driven hepcidin synthesis in the liver [40], hepatic hepcidin synthesis and, in return, systemic hepcidin levels may be reduced by PHZ hemolysis. Because both systemic and renal hepcidin are thought to be involved in renal protection, the significant reduction in systemic plasma hepcidin levels in Wt PHZ mice might have further aggravated renal injury in Wt PHZ mice compared to KO PHZ mice. Therefore, with the current experimental model, we were unable to differentiate between the effects of local renal hepcidin and systemic hepcidin on PHZ-mediated AKI. We summarize our hypothesis on systemic and local kidney iron handling during hemolysis-induced AKI in Figure 5. To truly determine the contribution of renal hepcidin to kidney protection, future studies should circumvent any interaction between systemic and local hepcidin while maintaining an as complete as possible kidney structure. Kidney organoids have already been used successfully to model renal diseases and screen for therapies [41]. Using renal organoids, the role of hepcidin in physiology and during heme-induced injury could be studied on an organ level. To date, no in vivo studies have been performed in which the effect of systemic and renal hepcidin could be distinguished. 

In line with our in vitro data and previous observations in hemoglobin-treated mice and mCCD_cl11_ cells [8], we expected a significant increase in renal *Hamp1* mRNA expression due to the PHZ-induced hemolytic kidney injury in Wt mice. Although *HO-1*, *TNFα,* and *IL-6* mRNA expression levels in PHZ Wt mice at D4 indicated renal injury, the *Hamp1* mRNA expression levels were not increased and even significantly lowered compared to control mice. A potential explanation could be that we missed the optimal time window in which renal hepcidin exerts its suggested protective effects. Previously, we observed a significant increase in renal *Hamp1* mRNA expression in C57Bl/6 mice injected with hemoglobin 24 h after their last hemoglobin injection [8]. Here, we used PHZ to more adequately mimic hemolysis. In contrast to the previous hemoglobin injection experiments, we waited an additional 24 h after the last PHZ injection to let the hemolysis set in, before we started the 24 h urine collection. Previous work has demonstrated that chronic in vitro exposure of proximal tubule epithelial cells (ciPTECs) to ferric iron resulted in Nrf2 exhaustion [23]. The longer in vivo incubation time, combined with our novel data on Nrf2-mediated hepcidin induction, might explain the lack of hepcidin induction observed at D4 after PHZ administration. 

Evidence supporting a protective role of hepcidin during AKI is increasing, which implicates that stimulating local hepcidin synthesis could be a potential therapeutic strategy. Yet, our understanding of renal hepcidin handling should first be improved further by dissecting the contribution of both locally and systemically produced hepcidin in the protection against AKI and unravel the molecular mechanisms involved in detail. Such insights are essential to fully comprehend the potential (side) effects of hepcidin modulators in the context of AKI.

In our study, we were not able to dissect the exact role of renal hepcidin in protection against heme-mediated injury. In contrast, our results further indicate the complex entanglement of different iron regulatory mechanisms on local and systemic levels that are also intertwined. Our study did, however, contribute to the molecular understanding of renal hepcidin regulation by demonstrating that renal hepcidin synthesis is not triggered by inflammation, as in the liver, but primarily through the release of iron, mediated by the Nrf2 pathway. The discovery of organ-specific mechanisms in renal hepcidin regulation emphasizes the necessity to continue investigating local mechanisms as potential targets for future therapy. 

## 4. Methods

### 4.1. Cell Culture

The mCCD_cl1_ cell line was generated and cultured as described previously at 37 °C in a 5% (*vol*/*vol*) CO_2_ atmosphere [42]. For each experiment, cells were seeded with approximately 12,500 cells/cm^2^, using passage number 29–35. 

Cells were incubated in serum-free medium with IL-6 (10 and 50 ng/mL; Gibco, Thermo Fisher Scientific, Breda, The Netherlands PHC0066), iron (II) sulphide (FeS, 200 and 500 µM; Sigma-Aldrich, St. Louis, MI, USA, F8263), lipopolysaccharide (LPS, 5 µg/mL; Sigma-Aldrich, St. Louis, MI, USA, L3129), hemin (10 µM; Sigma-Aldrich, St. Louis, MI, USA, H9039), and/or trigonelline hydrochloride (1 µM; Sigma-Aldrich, St. Louis, MI, USA, T5509).

### 4.2. Protein Isolation and Immunoblotting

Cell pellets were lysed using RIPA buffer (0.15 M NaCl, 0.012 M Sodium Deoxycholate, 0.1% NP40, 0.1% SDS, 0.05 M Tris, pH 7.5, freshly supplemented with protease inhibitors (Complete mini, Roche, Basel, Switzerland)). Protein concentrations were determined using the Pierce BCA assay kit (Thermo Fisher Scientific, Breda, the Netherlands), according to the manufacturer’s protocol. The samples were separated using an SDS-PAGE gel, transferred to a nitrocellulose membrane, and incubated with primary antibody at 4 °C overnight. After a 1 h incubation of the secondary antibody at RT, proteins were visualized with an Odyssey fluorescence scanner. Both primary and secondary antibodies and subsequent dilutions are summarized in Supplemental Table S1.

To collect nuclear protein fractions, cells were resuspended in a mild lysis buffer (10 mM NaCl, 1.5 mM MgCl_2_, 0.2 mM EDTA, 270 mM sucrose, 0.1% NP-40, 20 mM Tris−HCl, pH 7.5, freshly supplemented with 1 mM DTT and protease inhibitors) and disrupted with a Douncer homogenizer. Subsequent lysates were centrifuged at 3200 *g* for 15 min at 4 °C. Cell pellets were collected by centrifugation and lysed with RIPA buffer. For all immunoblotting experiments, equal amounts of protein were loaded.

Control kidney and liver homogenates from untreated C57Bl/6 mice were used as positive controls in the IL-6Rα Western blot. These were obtained from previous experiments [8]. 

### 4.3. Luciferase Assay

The luciferase reporter assay was performed using the Dual-Luciferase^®^ Reporter Assay System (Promega Corporation, Leiden, the Netherlands). Cells were co-transfected using lipofectamine 2000 (Invitrogen, Thermo Fisher Scientific, Breda, The Netherlands) with a Renilla containing continuously expressed SV40 plasmid and a hepcidin (encoded by the *Hamp1* gene) promotor sequence (*Hamp*1420, forward primer CGGGGTACCGAACCCTGTCTCGAGCTGGAG and reverse primer CTAGCTAGCGCCTTCTGTTCTGCTGTGCAG) coupled to a Firefly luciferase plasmid, respectively. Lipofectamine, SV40, and *Hamp*1420 were diluted in OptiMEM (Gibco) to 0.5, 0.02, and 0.3 μg, respectively. Cells were incubated with 100 µL of antibiotics free, 2% fetal calf serum medium, and 75 µL of lipofectamine mixture at 37 °C. After 6 h, culture medium was refreshed and after a total of 48 h post transfection, cells were incubated with either hemin with or without trigonelline, LPS, or IL-6 for 4 h or with FeS for 16 h. Luminescence was measured according to the manufacturer’s instructions using the VICTOR Multilabel plate reader (Perkin Elmer, Rotterdam, the Netherlands). Luciferase activity was quantified as the ratio of luminescence between *Hamp*1420 and SV40. 

### 4.4. Animal Studies

All experiments were approved by the local Animal Welfare Committee of the Radboudumc (DEC 2015-0084) in accordance with the guidelines of the Principles of Laboratory Animal Care (National Institutes of Health, Washington, DC, USA). Pax8-rtTA/LC-1 mice (University of Aachen, Aachen, Germany [43]) were crossbred with Hepcidin^flox/flox^ mice (INSERM, Paris, France [44]) to generate inducible kidney-specific hepcidin KO mice (Pax8-rtTA/LC-1/hepcidin^flox/flox^). Pax8-rtTA/LC-1/hepcidin^wt/wt^ mice were used as controls.

All mice (*n* = 30) were given Doxycycline ad libitum in the drinking water (1 g Doxycycline and 50 g sucrose/L water) for 21 days to induce the KO, followed by an 11 day washout period. For phenotyping, mice were subsequently placed in metabolic cages for 24 h urine collection, with pulverized standard chow and water ad libitum, followed by terminal blood collection.

For hemolysis experiments, both hepcidin^wt^ (*n* = 10) and hepcidin^flox^ (*n* = 10) were injected on two consecutive days with phenylhydrazine (PHZ, 60 mg/kg body weight) via i.p. injection. Hepcidin^wt^ mice (*n* = 10) were injected with saline as a control. On day 3 or day 9, the mice were placed in a metabolic cage for urine collection and sacrificed afterwards on day 4 or day 10 (*n* = 5 per timepoint) by terminal blood sampling. Protease inhibitors (Complete Mini; Roche Diagnostics, Basel, Switzerland) were added to urine samples, and plasma was collected in lithium-heparin tubes. Tissues were partly snap-frozen in liquid N_2_ and stored in formalin for immunohistochemistry. The urine creatinine concentration was determined using the assay kit based on the Jaffé method from Labor & Technik (LT-SYS 0251).

### 4.5. Immunohistochemistry

Tissue sections were embedded in paraffin, and 4-µm sections were mounted on APES-covered glass slides. Routine Perls Prussian Blue staining was performed by the pathology department (Radboudumc, Nijmegen, the Netherlands). Images were taken using the VisionTekDigital Microscope (Sakura). ImageJ was used to determine the percentage of the entire renal section positive for Perls iron staining.

### 4.6. ELISA

The concentrations of KIM-1 were determined in mouse urine samples using the DuoSet ELISA Development Kit (R&D Systems, Minneapolis, MN, USA DY1817) according to the manufacturer’s protocol.

### 4.7. Genotyping

Ear cuts were used for DNA extraction using the Direct PCR Lysis buffer (EAR, Viagen Biotech, Los Angeles, CA, USA) according to manufacturer’s protocol. PCRs were performed for *Cre*, *rtTA*, *LacZ,* and floxed *Hamp*. The primers are described in Supplementary Table S2. 

### 4.8. DNA Isolation and PCR

Kidney DNA was isolated with TRIzol^TM^ (Thermo Fisher Scientific), according to the manufacturer’s protocol, and concentrations were determined with the NanoDrop 2000 (Thermo Fisher Scientific). PCRs were performed for *Hamp* to ensure its deletion. Primers are described in Supplementary Table S3. 

### 4.9. Plasma and Urine Hepcidin Analysis

Plasma and urine hepcidin levels were determined as described previously [15]. Briefly, to a 25 µL plasma sample, 50 µL of acetonitrile (ACN) and 5 µL of internal standard (0.1 mM synthetic hhep-24; custom made; Peptide International, Louisville, KY, USA) were added. The solution was mixed and centrifuged at 27,500× *g* for 5 min. Of the prepared supernatant, 1.5 µL were spotted onto an MSP 96 Polished Steel Target Plate (Bruker Daltonics, Bremen, Germany) followed by 1.5 µL of energy-absorbing matrix (5 mg/mlacyano-4-hydroxy cinnamic acid; Bruker Daltonics). The sample and matrix were dried in an N2 atmosphere and measured using matrix-assisted laser desorption/ionization time-of-flight mass spectrometry (Bruker Daltonics). The concentration of mouse hepcidin was calculated by comparing its mass peak height with that of the internal standard, which had a final concentration of 10 nM. 

### 4.10. Plasma and Urine Non-Heme Iron Assay 

Plasma non-heme iron levels were determined using the Direct Ferene assay from Biolabo (92108) according to the manufacturer’s instructions.

Total non-heme iron levels were measured in urine using the bathophenanthroline assay (adapted from Torrance and Bothwell) [45].

### 4.11. RNA Isolation and qPCR

RNA was isolated with TRIzol^TM^ (Thermo Fisher Scientific), according to the manufacturer’s protocol, and concentrations were determined with the NanoDrop 2000 (Thermo Fisher Scientific). Reverse transcription PCR was performed in a 25 μL reaction volume with 1 μg of RNA, 4 μL of 5× first strand buffer, 1 μL of 12.5 mM dNTPs, 2.04 μL of 2.5 μg/μL random primers, 2 μL of 0.1 M DDT, 1 μL of M-MLV (all Thermo Fisher Scientific), and 0.5 μL of RNAsin (Promega Corporation) using the Applied Biosystems Thermal Cycler 2720. The PCR program included 10 min at 20 °C, 45 min at 42 °C, and 10 min at 95 °C. Quantitative PCR was performed with 4 μL of cDNA (10 ng/mL), 10 μL of SYBR Green master mix (Applied Biosystems, Thermo Fisher Scientific, Breda, the Netherlands), and 6 μL of primer mix (containing 1 μM forward primer and 1 μM reverse primer) using the Bio-Rad CFX96. The program consisted of 7 min at 95 °C, followed by 40 cycles of 15 s at 95 °C and 1 min at 60 °C, and 10 min at 95 °C. Fold change values were calculated relative to the housekeeping gene using the ΔΔCt formula. The primers used are summarized in Supplementary Table S4.

### 4.12. Statistical Analysis

Data are presented as mean ± SEM using GraphPad Prism 5.03 software (GraphPad Software, La Jolla, CA, USA). Results were analyzed by one-Way ANOVA with Bonferroni’s post test or a Student’s *t*-test, when appropriate. Differences were considered statistically significant with a *p* < 0.05. 

## Data Availability

The raw data supporting the conclusions of this article will be made available by the authors, without undue reservation.

## Supplementary Materials

The following are available online at https://www.mdpi.com/article/10.3390/ijms23031352/s1.

## References

[1] Uchino S., Kellum J.A., Bellomo R., Doig G.S., Morimatsu H., Morgera S., Schetz M., Tan I., Bouman C., Macedo E. (2005). Acute renal failure in critically ill patients: A multinational, multicenter study. JAMA.

[2] Mao H., Katz N., Ariyanon W., Blanca-Martos L., Adybelli Z., Giuliani A., Danesi T.H., Kim J.C., Nayak A., Neri M. (2014). Cardiac surgery-associated acute kidney injury. Blood Purif..

[3] Vercaemst L. (2008). Hemolysis in cardiac surgery patients undergoing cardiopulmonary bypass: A review in search of a treatment algorithm. J. Extra-Corpor. Technol..

[4] Moat N.E., Evans T.E., Quinlan G.J., Gutteridge J.M. (1993). Chelatable iron and copper can be released from extracorporeally circulated blood during cardiopulmonary bypass. FEBS Lett..

[5] Schaer D.J., Buehler P.W., Alayash A.I., Belcher J.D., Vercellotti G.M. (2013). Hemolysis and free hemoglobin revisited: Exploring hemoglobin and hemin scavengers as a novel class of therapeutic proteins. Blood.

[6] Haase M., Bellomo R., Haase-Fielitz A. (2010). Novel biomarkers, oxidative stress, and the role of labile iron toxicity in cardiopulmonary bypass-associated acute kidney injury. J. Am. Coll. Cardiol..

[7] Gburek J., Verroust P.J., Willnow T.E., Fyfe J.C., Nowacki W., Jacobsen C., Moestrup S.K., Christensen E.I. (2002). Megalin and cubilin are endocytic receptors involved in renal clearance of hemoglobin. J. Am. Soc. Nephrol..

[8] van Swelm R.P.L., Vos M., Verhoeven F., Thevenod F., Swinkels D.W. (2018). Endogenous hepcidin synthesis protects the distal nephron against hemin and hemoglobin mediated necroptosis. Cell Death Dis..

[9] Nath K.A. (2014). Heme oxygenase-1 and acute kidney injury. Curr. Opin. Nephrol. Hypertens..

[10] Kartikasari A.E.R., Wagener F.A.D.T.G., Yachie A., Wiegerinck E.T.G., Kemna E.H.J.M., Swinkels D.W. (2009). Hepcidin suppression and defective iron recycling account for dysregulation of iron homeostasis in heme oxygenase-1 deficiency. J. Cell. Mol. Med..

[11] Billings F.T., Ball S.K., Roberts L.J., Pretorius M. (2011). Postoperative acute kidney injury is associated with hemoglobinemia and an enhanced oxidative stress response. Free Radic. Biol. Med..

[12] Ho J., Reslerova M., Gali B., Gao A., Bestland J., Rush D.N., Nickerson P.W., Rigatto C. (2011). Urinary hepcidin-25 and risk of acute kidney injury following cardiopulmonary bypass. Clin. J. Am. Soc. Nephrol..

[13] Prowle J.R., Ostland V., Calzavacca P., Licari E., Ligabo E.V., Echeverri J.E., Bagshaw S.M., Haase-Fielitz A., Haase M., Westerman M. (2012). Greater increase in urinary hepcidin predicts protection from acute kidney injury after cardiopulmonary bypass. Nephrol. Dial. Transplant..

[14] Haase-Fielitz A., Mertens P.R., Plass M., Kuppe H., Hetzer R., Westerman M., Ostland V., Prowle J.R., Bellomo R., Haase M. (2011). Urine hepcidin has additive value in ruling out cardiopulmonary bypass-associated acute kidney injury: An observational cohort study. Crit. Care.

[15] van Swelm R.P., Wetzels J.F., Verweij V.G., Laarakkers C.M., Pertijs J.C., van der Wijst J., Thévenod F., Masereeuw R., Swinkels D.W. (2016). Renal Handling of Circulating and Renal-Synthesized Hepcidin and Its Protective Effects against Hemoglobin-Mediated Kidney Injury. J. Am. Soc. Nephrol..

[16] Kulaksiz H., Theilig F., Bachmann S., Gehrke S.G., Rost D., Janetzko A., Cetin Y., Stremmel W. (2005). The iron-regulatory peptide hormone hepcidin: Expression and cellular localization in the mammalian kidney. J. Endocrinol..

[17] Scindia Y., Dey P., Thirunagari A., Liping H., Rosin D.L., Floris M., Okusa M.D., Swaminathan S. (2015). Hepcidin Mitigates Renal Ischemia-Reperfusion Injury by Modulating Systemic Iron Homeostasis. J. Am. Soc. Nephrol..

[18] Kemna E., Pickkers P., Nemeth E., van der Hoeven H., Swinkels D. (2005). Time-course analysis of hepcidin, serum iron, and plasma cytokine levels in humans injected with LPS. Blood.

[19] Lim P.J., Duarte T.L., Arezes J., Garcia-Santos D., Hamdi A., Pasricha S.-R., Armitage A.E., Mehta H., Wideman S., Santos A.G. (2019). Nrf2 controls iron homeostasis in haemochromatosis and thalassaemia via Bmp6 and hepcidin. Nat. Metab..

[20] Suzuki T., Yamamoto M. (2015). Molecular basis of the Keap1-Nrf2 system. Free Radic. Biol. Med..

[21] Bayele H.K., Balesaria S., Srai S.K.S. (2015). Phytoestrogens modulate hepcidin expression by Nrf2: Implications for dietary control of iron absorption. Free Radic. Biol. Med..

[22] Liu J., Tan Y., Yang B., Wu Y., Fan B., Zhu S., Song E., Song Y. (2020). Polychlorinated biphenyl quinone induces hepatocytes iron overload through up-regulating hepcidin expression. Environ. Int..

[23] van Raaij S.E.G., Masereeuw R., Swinkels D.W., van Swelm R.P.L. (2018). Inhibition of Nrf2 alters cell stress induced by chronic iron exposure in human proximal tubular epithelial cells. Toxicol. Lett..

[24] Gozzelino R., Jeney V., Soares M.P. (2010). Mechanisms of cell protection by heme oxygenase-1. Annu. Rev. Pharm. Toxicol..

[25] Muckenthaler M.U., Galy B., Hentze M.W. (2008). Systemic Iron Homeostasis and the Iron-Responsive Element/Iron-Regulatory Protein (IRE/IRP) Regulatory Network. Annu. Rev. Nutr..

[26] Nezu M., Suzuki N., Yamamoto M. (2017). Targeting the KEAP1-NRF@ system to prevent kidney disease progression. Am. J. Nephrol..

[27] Zheng H., Whitman S.A., Wu W., Wondrak G.T., Wong P.K., Fang D., Zhang D.D. (2011). Therapeutic potential of Nrf2 activators in streptozotocin-induced diabetic nephropathy. Diabetes.

[28] Ma Q., Battelli L., Hubbs A.F. (2006). Multiorgan autoimmune inflammation, enhanced lymphoproliferation, and impaired homeostasis of reactive oxygen species in mice lacking the antioxidant-activated transcription factor Nrf2. Am. J. Pathol..

[29] Nezu M., Souma T., Yu L., Suzuki T., Saigusa D., Ito S., Yamamoto M. (2017). Transcription factor Nrf2 hyperactivation in early-phase renal ischemia-reperfusion injury prevents tubular damage progression. Kidney Int..

[30] Pergola P.E., Raskin P., Toto R.D., Meyer C.J., Huff J.W., Grossman E.B., Ruiz S., Audhya P., Wittes J., Warnock D.G. (2011). Bardoxolone methyl and kidney function in CKD with type 2 diabetes. N. Engl. J. Med..

[31] Kerins M.J., Ooi A. (2018). The Roles of NRF2 in Modulating Cellular Iron Homeostasis. Antioxid. Redox Signal..

[32] Duarte T.L., Talbot N.P., Drakesmith H. (2020). NRF2 and Hypoxia-Inducible Factors: Key Players in the Redox Control of Systemic Iron Homeostasis. Antioxid. Redox Signal..

[33] Puntarulo S. (2005). Iron, oxidative stress and human health. Mol. Asp. Med..

[34] Dendooven A., van Oostrom O., van der Giezen D.M., Leeuwis J.W., Snijckers C., Joles J.A., Robertson E.J., Verhaar M.C., Nguyen T.Q., Goldschmeding R. (2011). Loss of endogenous bone morphogenetic protein-6 aggravates renal fibrosis. Am. J. Pathol..

[35] El-Achkar T.M., Dagher P.C. (2015). Tubular cross talk in acute kidney injury: A story of sense and sensibility. Am. J. Physiol. Ren. Physiol..

[36] Nemeth E., Rivera S., Gabayan V., Keller C., Taudorf S., Pedersen B.K., Ganz T. (2004). IL-6 mediates hypoferremia of inflammation by inducing the synthesis of the iron regulatory hormone hepcidin. J. Clin. Investig..

[37] Traykova-Brauch M., Schönig K., Greiner O., Miloud T., Jauch A., Bode M., Felsher D.W., Glick A.B., Kwiatkowski D.J., Bujard H. (2008). An efficient and versatile system for acute and chronic modulation of renal tubular function in transgenic mice. Nat. Med..

[38] Coffey R., Ganz T. (2018). Erythroferrone: An Erythroid Regulator of Hepcidin and Iron Metabolism. HemaSphere.

[39] Kapitsinou P.P., Liu Q., Unger T.L., Rha J., Davidoff O., Keith B., Epstein J.A., Moores S.L., Erickson-Miller C.L., Haase V.H. (2010). Hepatic HIF-2 regulates erythropoietic responses to hypoxia in renal anemia. Blood.

[40] Stoffel N.U., Lazrak M., Bellitir S., Mir N.E., Hamdouchi A.E., Barkat A., Moretti D., Aguenaou H., Zimmermann M.B. (2019). The opposing effects of acute inflammation and iron deficiency anemia on serum hepcidin and iron absorption in young women. Haematologica.

[41] Yousef Yengej F.A., Jansen J., Rookmaaker M.B., Verhaar M.C., Clevers H. (2020). Kidney Organoids and Tubuloids. Cells.

[42] Gaeggeler H.P., Gonzalez-Rodriguez E., Jaeger N.F., Loffing-Cueni D., Norregaard R., Loffing J., Horisberger J.-D., Rossier B.C. (2005). Mineralocorticoid versus glucocorticoid receptor occupancy mediating aldosterone-stimulated sodium transport in a novel renal cell line. J. Am. Soc. Nephrol..

[43] Tenten V., Menzel S., Kunter U., Sicking E.M., van Roeyen C.R., Sanden S.K., Kaldenbach M., Boor P., Fuss A., Uhlig S. (2013). Albumin is recycled from the primary urine by tubular transcytosis. J. Am. Soc. Nephrol..

[44] Zumerle S., Mathieu J.R., Delga S., Heinis M., Viatte L., Vaulont S., Peyssonnaux C. (2014). Targeted disruption of hepcidin in the liver recapitulates the hemochromatotic phenotype. Blood.

[45] Torrance J.D., Bothwell T.H. (1968). A simple technique for measuring storage iron concentrations in formalinized liver samples. S. Afr. J. Med. Sci..

