# Supplementary material for: Investigating the Molecular Mechanisms of Renal Hepcidin Induction and Protection upon Hemoglobin-Induced Acute Kidney Injury
Source: Int J Mol Sci. 2022 Jan 25;23(3):1352. doi: 10.3390/ijms23031352 (PMC8835743; doi:10.3390/ijms23031352)
Supplement: Supplementary file 1 [file ijms-23-01352-s001.zip › ijms-1490691-supplementary-done.pdf]

International Journal of
Molecular Sciences

MDPI

Supplementary Material

Supplementary Table S1. Primary and secondary antibodies used for immunoblotting.

Protein	Primary Antibody	Dilution	Secondary Antibody	Dilution
Nrf2	Abcam 31163	1:500	Invitrogen A21109	1:20,000
NQO1	Abcam 34173	1:1000	Invitrogen A21109	1:20,000
ß-actin	Sigma A5441	1:100,000	Rockland 21031	1:20,000
Histone H3	Abcam 24834	1:1000	Rockland 21031	1:20,000
IL-6Rα	R&D Research AF1830	1:500	Thermo Fischer A27020	1:1000

Supplementary Table S2. Primers used for genotyping.

Gene	Forward primer (5′-3′)	Reverse primer (5′-3′)
Cre	GCATAACCAGTGAAACAGCATTGCTG	GGACATGTTCAGGGATCGCCAGGCG
rtTA	AATCGAGATGCTGGACAGGCATCAT-ACCCA	GGCATAGAATCGGTGGTAGGTGTCTCTCTT
LacZ	TTC ACT GGC CGT CGT TTTACAACG TCG TGA	ATG TGA GCG AGTAAC AAC CCG TCG GAT TCT
Hamp floxed	GGT GTC TAG GTT TTT TAC CCT	GAA GGC AAA GGC AAA TGA TC
β-actin	GCTATGCTCTCCCTCACGCCA	CTCTTTGATGTCACGCACGAT

Supplementary Table S3. Primers used for PCR after DNA isolation.

Gene	Forward primer (5′-3′)	Reverse primer (5′-3′)
Hamp	GAAGGCAAAGGCAAATGAT	GCAGCACCACCTATCTCCAT
HPRT	GCTGGTGAAAAGGACCTCTCG	CCACAGGACTAGAACACCTGC

Supplementary Table S4. Primers used for quantitative PCR.

**Gene**	**Forward primer (5′-3′)**	**Reverse primer (5′-3′)**
β-actin	GCTATGCTCTCCCTCAC-GCCA	CTCTTTGATGTCACGCAC-GAT
HPRT	GCTGGTGAAAAGGAC-CTCTCG	CCACAGGACTAGAACAC-CTGC
Hamp1	TTGCGATAC-CAATGCAGAAG	GGATGTGGCTCTAGGC-TATGTT
HO-1	CCTCACTGGCAG-GAAATCAT	CCAGAG-TGTTCATTCGAGCA
Keap1	ACGTCCTCGGAGGC-TATGAT	GGGTCACCTCAC-TCCAGGTA
GCLM	AGTTGACATGG-CATGCTCCG	CCATCTTCAATCG-GAGGCGA
TXNRD1	CAGCCCTGAA-GCCGAACAAA	GTCATAGGACCCAGGGG-GAT
NQO1	GGTAGCGGCTCCATGTACTC	CGCAGGATGCCAC-TCTGAAT
L-ferritin	GAAACTCATCAAGAA-GATGGGCA	GCTGGTTGTGGCCCCGC
IL-6	GAGGATACCAC-TCCCAACAGACC	AAGTGCATCATCGTT-GTTCATACA
TfR1	CGCTTTGGGTGCTGGTG	GGG-CAAGTTTCAACAGAA-GACC
